# Ym155 Induces Oxidative Stress-Mediated DNA Damage and Cell Cycle Arrest, and Causes Programmed Cell Death in Anaplastic Thyroid Cancer Cells

**DOI:** 10.3390/ijms22041961

**Published:** 2021-02-16

**Authors:** Qinqin Xu, Ryan P. Mackay, Adam Y. Xiao, John A. Copland, Paul M. Weinberger

**Affiliations:** 1 Departments of Otolaryngology, Head & Neck Surgery, LSU Health Shreveport, 1501 Kings Highway, Shreveport, LA 71103, USA; qxu1@lsuhsc.edu (Q.X.); rmacka@lsuhsc.edu (R.P.M.); 2 Departments of Molecular and Cellular Physiology, LSU Health Shreveport, 1501 Kings Highway, Shreveport, LA 71103, USA; axiao@lsuhsc.edu; 3 Department of Cancer Biology, Mayo Clinic, Jacksonville, FL 32224, USA; Copland.John@mayo.edu

**Keywords:** anaplastic thyroid cancer, YM155, DNA damage, cell cycle arrest, apoptosis

## Abstract

Anaplastic thyroid cancer (ATC) is one of the most lethal malignancies with a median survival time of about 4 months. Currently, there is no effective treatment, and the development of new therapies is an important and urgent issue for ATC patients. YM155 is a small molecule that was identified as the top candidate in a high-throughput screen of small molecule inhibitors performed against a panel of ATC cell lines by the National Cancer Institute. However, there were no follow-up studies investigating YM155 in ATC. Here, we determined the effects of YM155 on ATC and human primary benign thyroid cell (PBTC) survival with alamarBlue assay. Our data show that YM155 inhibited proliferation of ATC cell lines while sparing normal thyroid cells, suggesting a high therapeutic window. YM155-induced DNA damage was detected by measuring phosphorylation of γ-H2AX as a marker for DNA double-strand breaks. The formamidopyrimidine-DNA glycosylase (FPG)-modified alkaline comet assay in conjunction with reactive oxygen species (ROS) assay and glutathione (GSH)/glutathione (GSSG) assay suggests that YM155-mediated oxidative stress contributes to DNA damage. In addition, we provide evidence that YM155 causes cell cycle arrest in S phase and in the G2/M transition and causes apoptosis, as seen with flow cytometry. In this study, we show for the first time the multiple effects of YM155 in ATC cells, furthering a potential therapeutic approach for ATC.

## 1. Introduction

Anaplastic thyroid cancer (ATC) only represents 1–2% of all thyroid cancers, however, ATC accounts for 40–50% of all thyroid cancer deaths [1,2]. ATC remains a clinical challenge due to its highly aggressive characteristics [1,3,4]. ATC metastasizes quickly to other organs with a median survival time of 4 months following diagnosis. Indeed, Surveillance, Epidemiology, and End Results (SEER) data show that 80% of ATC patients do not survive longer than a year after diagnosis, and the 5-year overall survival rate is less than 5% [3,4,5]. This is partially due to a lack of effective treatment. All current therapeutics fail to significantly prolong ATC patients’ survival. The development of an effective drug is important and urgent for ATC patients.

YM155, also known as sepantronium bromide, was identified as the top candidate in a high-throughput screen of 3282 chemotherapy and small molecule inhibitors performed against a panel of ATC cell lines [6]. However, few follow-up studies have been performed to test the effects of YM155 in ATC or to uncover additional mechanisms of action. YM155 is a novel small molecule that was first identified as a survivin inhibitor. However, clinical trials evaluating YM155 in solid tumors (not including ATC) failed to demonstrate meaningful efficacy, and there was no association between survivin expression and response to YM155 [7,8,9]. Recent studies have shown that DNA damage occurred with YM155 treatment in different cell lines including acute lymphoblastic leukemia cells and breast cancer [10,11]. YM155 also targets interleukin enhancer-binding factor 3/NF110 in PC-3, Calu-6, and HeLa cells [12]. A recent report of YM155 by Hong et al. [13] performed in an in vitro study of lung cancer showed that topoisomerase IIα (Top2α) was a target of YM155. Uncovering the effects and mechanism of YM155 in ATC is essential to predict patient response to therapy, identify synergistic drug combinations, and minimize toxicity.

In the present study, we focused on testing the effects of YM155 in ATC cells including anti-proliferative activity, induction of DNA damage, cell cycle arrest, and apoptosis. We also tested the effect of YM155 on primary benign thyroid cells (PBTCs). Our data show that YM155 inhibited proliferation of ATC cell lines while sparing normal thyroid cells and suggests a high therapeutic window.

## 2. Results

### 2.1. Effect of YM155 on ATC and Benign Thyroid Cell Growth

**Figure 1:**
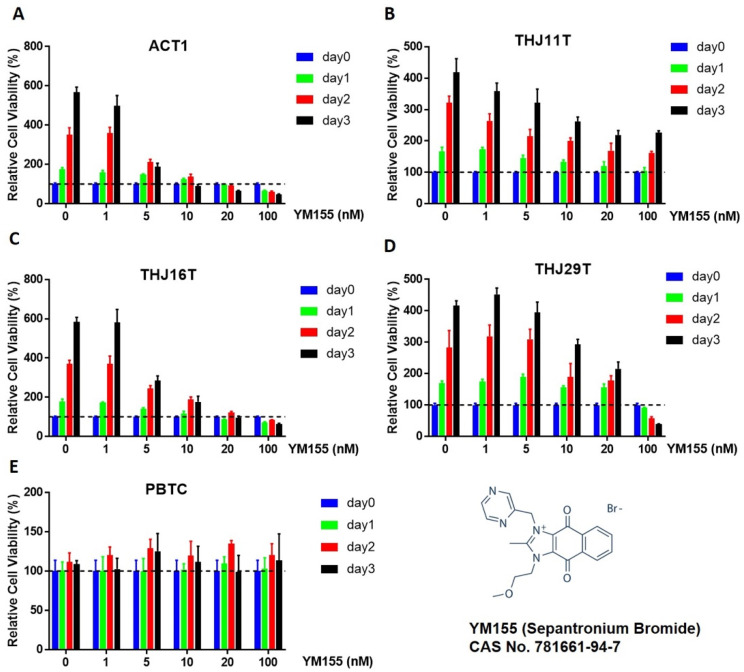
YM155 inhibited proliferation of anaplastic thyroid cancer (ATC) cell lines while sparing normal thyroid cells. ATC cell lines (**A**–**D**) and primary benign thyroid cells (PBTCs) (**E**) were treated with varying concentrations of YM155 and incubated for 0, 1, 2, or 3 days. Cell viability was measured using the alamarBlue (Bio-Rad, Oxford, UK) assay. ATC cell lines ACT1 (**A**) and THJ16T (**C**) were fast growing and showed dramatic responses to YM155 treatment, even at low doses. ATC cell lines THJ11T (**B**) and THJ29T (**D**) demonstrated slower growth, comparatively, and were less responsive to YM155 treatment, with complete proliferation inhibition occurring only at higher doses in THJ29T cells and incomplete proliferation inhibition in THJ11T. (**E**) Primary benign thyroid cells exhibited slow growth and were unaffected by YM155 at all concentrations and times. IC_50_ for each cell line: ACT1 = 3.24 nM, THJ16T = 5.102 nM, THJ11T = 73.387 nM, THJ29T =18.6433 nM.

It has been previously demonstrated that YM155 inhibits proliferation in ATC cell lines THJ16T and THJ29T [6]. To confirm and extend these findings, we used alamarBlue assay as an indicator of proliferation in cell lines used in the previous study (THJ16T and THJ29T) along with two other ATC cell lines (ACT1 and THJ11T) and human primary benign thyroid cells (PBTCs) collected intraoperatively. PBTC identity and differentiation were verified by detection of thyroid-specific markers (Figure S1). Nanomolar concentrations of YM155 inhibited proliferation in all four ATC cell lines in a dose- and time-dependent manner (Figure 1A–D). IC_50_ for each cell line was calculated as ACT1 3.24 nM, THJ16T 5.102 nM, THJ29T 18.6433 nM, and THJ11T 73.387 nM, respectively. We also found that slow-growing PBTCs were unaffected by YM155 treatment, even when treatment was extended up to 6 days (Figure 1E). Interestingly, the sensitivity of YM155 was highly variable between different ATC cell lines. The proliferation of ACT1 and THJ16T cells was significantly inhibited at 10 nM with near-total loss of proliferation by day 3 while THJ11T and THJ29T cells showed only modest inhibition at this concentration compared to untreated controls.

### 2.2. YM155 Selectively Induced DNA Damage in ATC Cells

**Figure 2:**
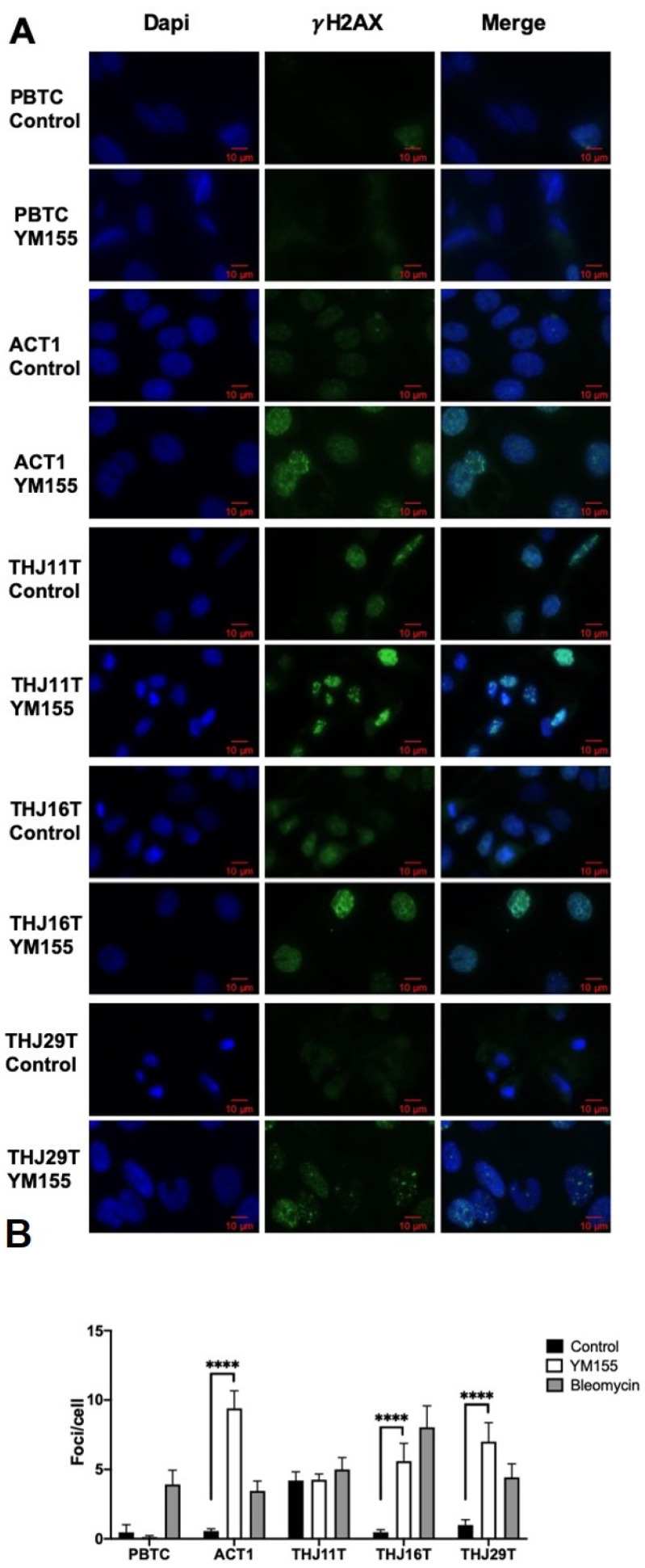
YM155 induced DNA damage in ATC cells while human primary benign thyroid cells were unaffected. ATC cells ACT1, THJ16T, and THJ29T showed increases 4hosphorpho-histone H2AX (γ-H2AX), a DNA repair factor and marker for double-strand DNA breaks, after treatment with 10 nM YM155 for 24 h. Cell line THJ11T exhibited elevated levels of γ-H2AX at baseline, which were not significantly increased by YM155 treatment. PBTCs, which exhibited DNA damage when treated with the positive control bleomycin, showed no evidence of DNA damage with YM155 treatment. (**A**) Examples of pictures captured by fluorescent microscopy. (**B**) Foci were counted using JQuantPlus [15,16]. γ-H2AX, a count level data, was analyzed using negative binomial regression and reported with mean and 95% confidence interval. Corresponding *p*-value is associated with the regression. **** indicates *p* < 0.0001.

YM155 inhibits ATC cells growth; however, the mechanism remains unclear. Because of the fast growth of ATC cells compared to PBTCs and the difference in response to YM155, we hypothesized that YM155 may induce DNA damage in ATC cells. Phosphorylated histone H2AX (γ-H2AX) is an established surrogate marker for DNA double-strand breaks, with a tight correlation between foci per cell and the number of double-strand breaks [14]. We measured γ-H2AX levels in PBTCs and ATC cells, with or without YM155 treatment. As a positive control, bleomycin increased γ-H2AX in all cell lines including PBTCs. YM155 significantly increased γ-H2AX in ATC cell lines ACT1, THJ16T, and THJ29T, while PBTCs were unaffected (Figure 2). Additionally, cell line THJ11T had high γ-H2AX levels at baseline that were not significantly increased with YM155 treatment. Using negative binomial regression to predict number of foci on the basis of YM155 treatment, we found that 24-hour treatment with 10 nM YM155 resulted in 17.0 times more foci than control for ACT1 cells (95% CI: 12.39 to 23.34, *p* < 0.0001), 11.8 times more foci in THJ16T cells (95% CI: 7.65 to 18.22, *p* < 0.001), and 7.1 times more in THJ29T cells (95% CI: 4.628 to 10.762, *p* < 0.0001). Notably, ATC cell lines ACT1 and THJ16T, which were more sensitive to YM155 in the cell viability assay, also exhibited greater increases in γ-H2AX.

### 2.3. YM155 Increased Oxidative Stress in ATC Cells

**Figure 3:**
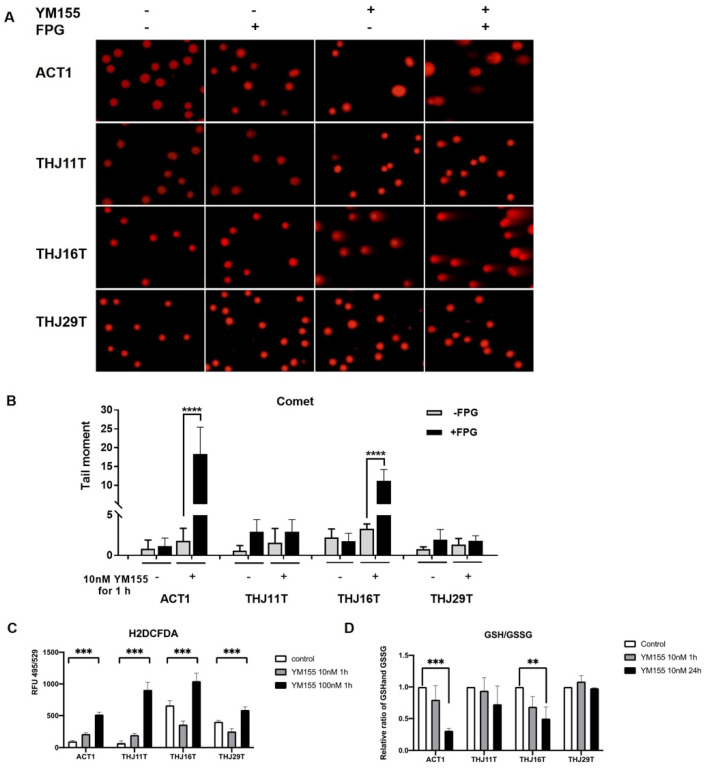
YM155 increased oxidative stress in THJ16T and ACT1. (**A**) Representative comet assay images. Alkaline comet assay was used to measure single-strand DNA breaks after treatment with 10 nM YM155 for 1 h with or without formamidopyrimidine-DNA glycosylase (Fpg) treatment. Tail moment increased significantly in Fpg+ ACT1 and THJ16T cells, with lower tail moment in Fpg- samples, suggesting that YM155 induces oxidative DNA damage in 1 h. (**B**) Quantification of tail moment with OpenComet plugin for ImageJ. (**C**) Effect of YM155 in reactive oxygen species (ROS) assay. Oxidation of H2DCF by intracellular ROS yielded a highly fluorescent product, H2DCFDA, which was detected by microplate reader (Ex/Em 495/529 nm). ROS increased in ATC cells after 1-h treatment with 100 nM YM155. (**D**) Effect of YM155 in glutathione fluorometric assay. The OPA probe (o-phthalaldehyde) reacted with glutathione (GSH), generating fluorescence at Ex/Em 340/420 nm. To measure glutathione (GSSG), we added a GSH quencher to remove GSH, preventing reaction with OPA, and a reducing agent was then added to remove excess quencher and convert GSSG to GSH. Thus, GSSG can be specifically quantified. Concentration of GSH and GSSG was calculated on the basis of a GSH standard curve. Results were normalized to the control group. **** indicates *p* < 0.0001; *** indicates *p* < 0.001; ** indicates *p* < 0.01.

To determine whether YM155 increases oxidative stress in ATC, we performed the formamidopyrimidine-DNA glycosylase (Fpg)-modified alkaline comet assay. Cells were treated with Fpg (formamidopyrimidine-DNA glycosylase) before electrophoresis to generate breaks at the sites of oxidative pyrimidine damage including 8-oxoguanine [17]. As shown in Figure 3A,B, YM155 treatment increased comet tail length in cell lines ACT1 and THJ16T, and comet tails increased further with the addition of Fpg in ACT1 and THJ16T cells, suggesting that oxidative base damage contributes to YM155-induced DNA damage in these cells. We did not see obvious changes in THJ11T, consistent with our previous cell viability and γ-H2AX data. However, we failed to detect oxidative DNA damage in THJ29T, despite increases in DNA damage after 24-h treatment in the previous γ-H2AX assay. Oxidative stress is a result of abnormal accumulation of reactive oxygen species (ROS), mitochondrial dysfunction, reduced antioxidant capacity such as a decreased ratio of reduced glutathione (GSH) to oxidized glutathione (GSSG), or a combination of these factors [18]. Here, we also performed ROS assay and glutathione fluorometric assay to determine if oxidative stress was induced by YM155 in ATC cells. As shown in Figure 3C,D, all four ATC cell lines exhibited elevated ROS levels after 1-hour treatment with 100 nM YM155, while ACT1 and THJ16T cells showed a decreased ratio of GSH to GSSG.

### 2.4. YM155 Induced S Phase or G2/M Arrest in ATC Cells

**Figure 4:**
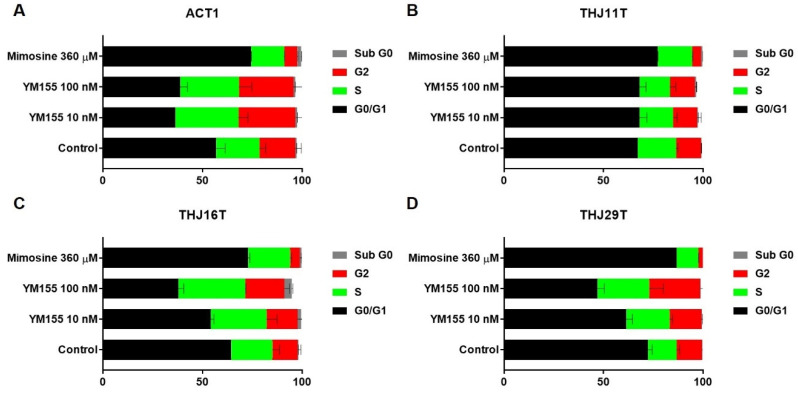
YM155 induced cell cycle arrested in ATC cells. ATC cell lines ACT1 (**A**), THJ11T (**B**), THJ16T (**C**), and THJ29T (**D**) treated with YM155 (10 nM), mimosine (360 µM), or control (1/1000 DMSO) for 24 h were fixed and processed, stained with propidium iodide (PI), and analyzed with flow cytometry. Cell cycle analysis calculated the proportion of cells in G0/G1, S, G2, and sub G0. Cells were arrested at S phase and G2/M with YM155 treatment, while the positive control mimosine caused stalling in G0/G1 phase, suggesting that YM155-induced DNA damage occurs during DNA replication.

Cell cycle arrest is a self-protective mechanism following DNA damage. Here, we performed cell cycle analyses in four ATC cell lines (ACT1, THJ11T, THJ16T, and THJ29T) after YM155 or mimosine treatment. As shown in Figure 4, cells were drastically stalled at S phase and G2/M transition after 10 or 100 nM YM155 treatment for 24 h in ACT1, THJ16T, and THJ29T, while all four cell lines arrested in G1 phase after treatment with mimosine, a drug that arrests dividing cells in the late G1 phase by inhibiting DNA replication initiation [19]. Cell cycle distribution of THJ11T (Figure 4B) was not markedly affected by YM155, which was reasonable since no DNA damage was detected in THJ11T. We detected increased activated/phosphorylated Chk1 (p-Chk1) after 10 nM YM155 treatment for 16 h in ATC1 and THJ16T (Figure S2), suggesting that YM155-induced DNA damage led to Chk1 activation followed by cell cycle arrest at S and G2/M phase.

### 2.5. YM155 Induced Apoptosis in ATC Cells ACT1 and THJ16T

**Figure 5:**
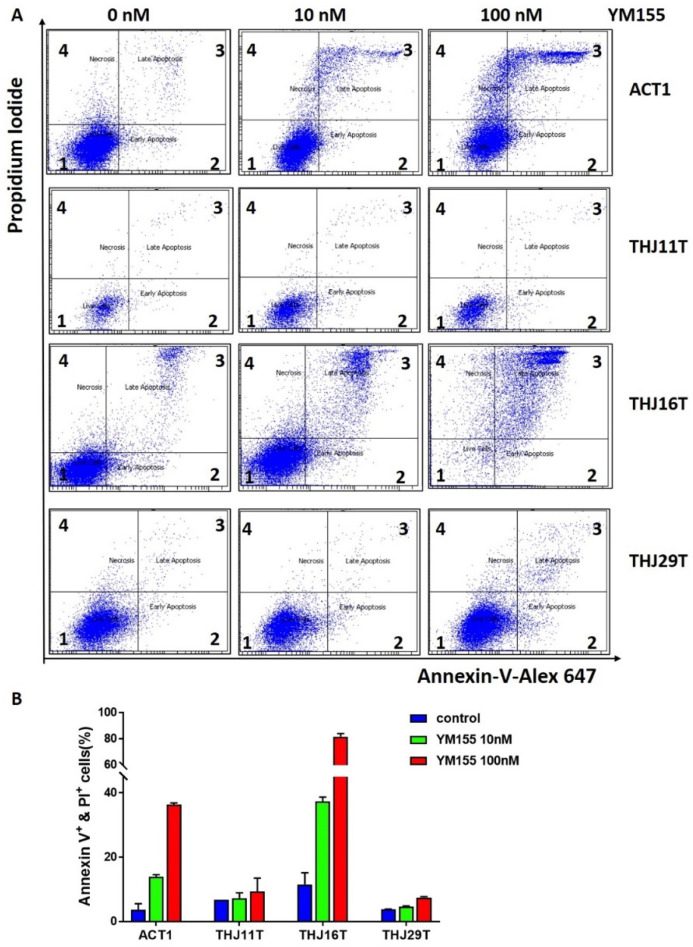
YM155 induced apoptosis in ATC cell lines THJ16T and ACT1. (**A**) Selected profiles of apoptosis analysis with Alexa Fluo 647 annexin V in ATC cells. The four quadrants 1, 2, 3, and 4 represent live cell, early apoptosis, late apoptosis, and necrosis, respectively. (**B**) The bar graph indicates the percent of apoptotic and necrotic cells in each group. Annexin V+ indicates annexin V-positive cells. PI+ indicates propidium iodide-positive cells.

DNA damage may also induce cell programmed death (apoptosis) when the damage is extensive. ATC cells were treated with either 10 nM YM155, 100 nM YM155, or 200 nM doxorubicin and analyzed with Alexa Fluor 647 Annexin V kit to determine the proportion of live cells, apoptotic cells, and necrotic cells. The classic chemotherapeutic drug doxorubicin failed to induce apoptosis in all four ATC cell lines at 200 nM concentration. Twenty-four-hour treatment with YM155 induced apoptosis in ACT1 and THJ16T but not in THJ11T or THJ29T (Figure 5).

## 3. Discussion

At present, there is no effective treatment for most ATC patients. All current therapies including surgery, radiation, and chemotherapy fail to significantly prolong ATC patients’ survival. ATC remains a clinical challenge due to its highly aggressive characteristics [1,3,4]. The most common driver mutations for anaplastic thyroid cancer occur in the *BRAF* or *RAS* genes, and drugs that target the BRAF kinase have had higher response rates than cytotoxic chemotherapy [20]. Targeted therapy appears promising against ATC; however, only 20–40% of ATCs harbor a *BRAF* mutation [21]. Immunotherapy could potentially be used against anaplastic thyroid cancer, as preclinical studies have shown that anaplastic thyroid tumors express high levels of the immune checkpoint protein programmed cell death protein 1 (PD-1) and its ligand, PD-L1 [1,22]. However, it takes time for the immune system to respond to drugs that inhibit PD1 and PD-L1. Anaplastic thyroid cancer patients are short of time since they usually present at advanced stages. Thus, development of effective pharmacologic therapies is important and urgent for ATC patients, and understanding the mechanism of action of potential drugs for ATC is extremely important for future clinical use.

Traditional approaches to drug discovery require a significant investment of time and funding. An emerging approach to developing therapies for rare or orphan cancers, such as ATC, instead exploits the multitude of established compounds that are already approved for clinical use or currently in clinical trials. YM155 is one of the most active agents selected in a previous screening of 3282 clinically approved drugs in ATC cell lines [6]. Of the 3282 drugs studied, 3182 (97%) lacked efficacy across three different ATC cell lines, matching clinical experience. Of the 3% showing efficacy, the top compound was the survivin inhibitor YM155, which was effective in killing all ATC cell lines tested at low nanomolar concentrations. In Mehta et al.’s study, the antitumor activity of YM155 was validated in an in vivo ATC metastases mouse model, demonstrating the potent anti-tumor activity of YM155 as well as a very favorable side effect profile (no significant difference in weight between treated and untreated mice, and no serious adverse effects were observed during the treatment period) [6].

Identifying the mechanism of action of YM155 treatment is essential to predict patient response to therapy, identify synergistic drug combinations, and minimize toxicity. The proposed mechanism of YM155 as a survivin inhibitor does not fully explain the activities of YM155 in other cancer studies. A negative clinical trial of YM155 in patients with non-small cell lung cancer showed no correlation between suppression of survivin and patient response to YM155 [8]. The target molecules of YM155 have remained unclear and studies appear to hinge on multiple synergistic downstream targets of YM155 beyond simple survivin inhibition [10,11,23,24,25,26].

Here, we discovered the effect of YM155 in several ATC cell lines. These ATC cell lines were chosen as the models for this study, since all cell lines included are well-characterized for identification of molecular mechanisms related to tumor biology and drug responsiveness [27,28]. Although our data suggest that YM155 inhibits cell growth in ATC cells, this did not occur in benign thyroid cells even at 10-fold higher doses, suggesting a high therapeutic window.

Interestingly, we also noticed that YM155 had different effects even among ATC cell lines. Cell lines ACT1 and THJ16T were very sensitive to YM155, while THJ11T and THJ29T showed limited response to YM155. In alamarBlue cell viability assay, ACT1 and THJ16T were significantly affected at 5 nM concentration of YM155, while no difference was seen in THJ11T and THJ29T until 10 nM or higher. Similar results were seen across time; an effect was more likely to be seen in THJ11T and THJ29T when extending treatment to 2 days or more. This may also help explain why ROS levels were increased in all four ATC cells with 100 nM YM155 treatment for 1 h, yet we were unable to detect a change in DNA damage in comet assay in these two cell lines.

DNA damage can be caused by endogenous oxidative stress or exogenous factors such as UV light or various chemicals agents. We performed γ-H2AX staining to determine if YM155 induces damage in ATC and found an increase in foci in ACT1, THJ16T, and THJ29T but not THJ11T. Importantly, YM155 had no effect on benign thyroid cells confirmed by cell viability data, lending confidence to the potential for clinical application. Several studies have reported increased DNA damage after YM155 treatment in several cancer cell lines [10,11,24,29,30,31]. It is uncertain whether YM155 causes DNA damage directly or indirectly [32]. We hypothesized that YM155 induces DNA damage by increasing cellular ROS, and we employed the Fpg-modified alkaline comet assay to explore YM155′s ability to cause oxidative DNA damage. Because Fpg augmented the increases in DNA damage seen with YM155 treatment in cell lines THJ16T and ACT1, these increases can be attributed to oxidative DNA damage. It remains unclear whether YM155 causes DNA damage indirectly by altering cellular redox signaling or directly through redox cycling that happens at its quinone group, or both [33]. Our results suggest that altered cellular ROS levels contribute to DNA damage with YM155 treatment.

Cell cycle arrest is one of the cellular responses to DNA damage to preserve genomic integrity. Checkpoints serve to monitor the order of events in the cell cycle and ensure that subsequent cell cycle events occur only after the completion of a prior event [34]. DNA damage causes cell cycle arrest either before DNA replication in G1, within DNA synthesis phase (S phase), at the S phase DNA damage checkpoint (SDDC), or before mitosis in G2 (G2/M checkpoint) [35,36]. Our cell cycle analysis suggests that DNA damage induced by YM155 happens during DNA replication (S phase) and G2/M stage rather than the regular cell growth and metabolic stage (G1). Chk1 and Chk2 are both activated upon DNA damage and can regulate cell division. Chk1 activity has been mostly implicated in the S phase cell cycle checkpoint and the G2/M transition [36,37,38]. DSBs are repaired by two major pathways: DNA non-homologous end joining (NHEJ) or homologous recombination (HR). NHEJ repairs DSBs in all cell cycle phases and represents the major pathway in G1, while HR functions in S and G2 [39]. The arrest in S and G2 phase also suggests YM155-mediated DNA damage may involve key proteins in HR or other proteins highly expressed in G2 such as DNA topoisomerase II-alpha (Top2a) [40].

Programmed cell death (apoptosis) is a cell-controlled process triggered by extreme alterations to cellular homeostasis. Initial signaling events, such as extensive irreparable DNA damage, set off cascades to activate caspases, leading to apoptosis. Several studies have reported that YM155 induced apoptosis in cancer cells such as glioma, HeLa, and prostate cancer cells [24,41,42]. In this study, YM155 induced apoptosis in ATC cancer cell lines ACT1 and THJ16T but not THJ11T or THJ29T, which is also consistent with the other data of this study.

Although the target molecules of YM155 in ATC have remained unclear, studies imply multiple synergistic downstream targets of YM155 exist beyond survivin. Here, we have confirmed and discovered previously unreported effects of YM155 in ATC. As a result, cell lines ACT1 or THJ16T represent good models to study the mechanism of action of YM155 in ATC. These results also suggest that YM155 may not be effective in all ATC patients. Further research could reveal components of the molecular signature of cell lines ACT1 or THJ16T that result in sensitivity to YM155. Similar profiles in ATC patients could allow for individualized therapy. Our lab is actively working on identifying the target of YM155 in THJ16T cells, which could then be used as a biomarker for the use of YM155 in ATC patients. These experiments will contribute to the generation of genetically engineered mouse models. Such models, with specific modifications in anaplastic thyroid cancer targets, could provide more accurate information for pre-clinical drug studies than traditional in vivo models such as the metastases mouse model. Future studies of YM155 in such models would strengthen current findings and allow for clinical studies in ATC patients.

## 4. Material and Methods

### 4.1. Cell Culture

Patient-derived xenograft ATC lines THJ11T, THJ16T, and THJ29T were obtained as gifts from Dr. J. Copland (Mayo Clinic, Jacksonville FL) [28], and patient-derived ATC line ACT1 was a gift from Dr. S. Ohata (Tokushima University, Tokushima, Japan) [27]. Cell lines were maintained in Dulbecco’s Modified Eagle Medium (DMEM) (+4.5 g/L glucose, L-glutamine, sodium pyruvate) supplemented with 10% heat-inactivated fetal bovine serum (Thermo Fisher Scientific, Waltham, MA, USA) and 1% Antibiotic-Antimycotic (AA) (Thermo Fisher Scientific, Waltham, MA, USA).

PBTCs were prepared following the protocol described by Wang et al. [41] with some modification. Fresh thyroid tissue was kept in culture medium on ice while being transferred to the laboratory. The tissue was cut into small pieces and aliquoted into vials with medium and stored in liquid nitrogen or used immediately. The fresh tissue was dissected and minced into fragments as small as possible using sterile scissors in a cell culture hood. After washing with 1× phosphate-buffered saline (PBS) containing 1% AA on a 40 µm nylon mesh (Thermo Fisher Scientific, Waltham, MA, USA), the tissue fragments were collected and transferred to 0.25% trypsin solution for overnight digestion in a 5% CO_2_ 37 °C incubator. Fragments were filtered and collected again on day 2. The fragments were digested with 2 mL trypsin with 50 µL (28.5 U/µL) Collagenase Type 2 (Worthington, Lakewood, NJ, USA) in an incubator for 2 h. The digested material was filtered and collected for the next step; the undigested tissue fragments were processed in the same manner one more time. The digested tissue was centrifuged at 1000 rpm for 5 min, and the pellet was added into 1 mL 1× red-blood cell lysis buffer (BioVision, Milpitas, CA, USA) at room temperature for 5 min. The cells were washed twice with PBS. Finally, the cells were counted using Cellometer Mini automated cell counter (Nexcelom Bioscience, Lawrence, MA, USA) and seeded to a density of 5 × 10^5^ cells per well in 6-well plates in 2 mL of Normal Thyroid Epithelial Cell Growth Medium (CHI Scientific, Maynard, MA, USA) with 10% fetal bovine serum (FBS). Thyroid cell markers TG, TPO, TSHR, FOXE1, NKX2-1, and PAX8 were used to confirm cell identity, and 18S rRNA was used as an internal control. The primer sequences are shown in Table S1.

### 4.2. AlamarBlue Assay

Cells were plated 4–8 × 10^3^ cells per well in 96-well plates. After 16 h, cells were treated with YM155 in various concentrations for 24, 48, or 72 h. Viability of cultured cells was analyzed using alamarBlue (Bio-Rad, Oxford, UK). Cells were incubated for 1 h after stimulation with alamarBlue according to the manufacturer’s instructions. Fluorescence was read at excitation 560 nm, emission 590 nm. To calculate differences in proliferation between treated and control cells, we used the following formula for relative viability (RV):

$$RV=\;\frac{Treated\;Sample_{Fl\;590}-background_{Fl\;590}\;}{Untreated\;Control_{Fl\;590}-\;background_{Fl\;590}}$$

where background is measured using media (without cells) plus alamarBlue.

### 4.3. Immunofluorescence

Immunofluorescent detection of phosphorylated histone H2AX at serine 139 (γ-H2AX) foci was used as a surrogate marker for formation of double-strand DNA breaks (DSBs). ATC cells were seeded on coverslips in a 24 well plate and treated with YM155 when 80% confluent. After 24 h treatment with 10 nM YM155, cells were washed with PBS (500 µL) for 5 min then fixed with 4% paraformaldehyde for 15 min, permeabilized with permeabilization buffer (20 nM HEPES, 50 mM NaCl, 3 mM MgCl_2_, 300 mM sucrose, 0.5% Triton X-100) for 7 min, washed with PBS (500 µL) for 5 min, blocked with 5% normal goat serum in PBS for 1 h, and incubated overnight at 4 °C with γ-H2AX (Ser139, 1:2000, MilliporeSigma, Burlington, MA, USA) antibody. For visualization, cells were incubated with AlexaFluor-488-conjugated rabbit anti-mouse secondary antibody (Cat. A-11017, 1:1000, Thermo Fisher Scientific) for 1 h and shielded from light. DAPI (1 µg/mL in PBS) was used as a nuclear counterstain. A minimum of 50 cells in each experimental group were imaged using an epifluorescent Olympus BX43 microscope(Olympus, Tokyo, Japan) with cooled CCD camera, and foci were counted using the software JQuantPlus, which is a further development of JCountPro [15,16] with the same parameters applied to all groups. Statistical analysis for γ-H2AX foci was performed using negative binomial regression. For comparisons of number of γ-H2AX foci (a count level data), a negative binomial regression with log link function and robust estimation was performed using SPSS Statistics version 25 (IBM, New York, NY, USA) to predict the number of foci based on YM155 treatment or Top2α siRNA 99. Results are reported using likelihood ratio with 95% confidence interval as well as the p value for the statistical model, and results are represented graphically as mean number of foci/cell ± confidence interval.

### 4.4. Fpg-Modified Alkaline Comet Assay

The Fpg-modified alkaline comet assay was performed as described by Xiao et al. [42] with slight modification. Cells with and without treatment were collected, washed with PBS, and suspended in 0.6% low melting point agarose (LMPA) at an appropriate concentration and placed onto slides. Slides were incubated in lysis buffer (100 mM Ethylenediaminetetraacetic acid (EDTA), 2.5 M NaCl, 10 mM, Tris-HCl, 1% Triton-X; pH 10) overnight at 4 °C and equilibrated in enzyme reaction buffer (40 mM HEPES, 0.1 M KCl, 0.6 mM EDTA, 0.2 mg/mL Bovine serum albumin (BSA); pH 8). Then, samples were either treated with 8 U/mL of Fpg (M0240S, NEB, Ipswich, MA, USA) in NEB buffer1 or in buffer1 without Fpg for 30 min at 37 °C. After separating DNA strands in alkaline buffer (0.3 M NaOH, 1 mM EDTA) for 20 min at 30 V and 300 mA, we placed slides in neutralization buffer (0.4 M Tris-HCl; pH 7.5) for 20 min to allow for DNA recondensation. Samples were dried overnight and stained with propidium iodide at 2 µg/mL. Slides were imaged using a Nikon Eclipse at 20X objective, and tail moment was calculated using OpenComet plugin for ImageJ. More than 50 cells were analyzed for each sample and the average tail moment with 95% CI was calculated.

### 4.5. ROS Assay

Reactive oxygen species (ROS) were detected using the Biovision kit (K936-250) (Milpitas, CA, USA) according to manufacturer’s protocol. Briefly, 1 × 10^4^ cells per well were seeded in a 96-well plate. The next day, media was removed, and adherent cells were washed in 100 μL of ROS Assay Buffer. They were then incubated in 100 μL of 1× ROS Label diluted in ROS Assay Buffer for 40 min at 37 °C in the dark followed by treatment with 100 μL medium containing 0, 10, or 100 nM YM155 for 1 h. Fluorescence intensity was measured at Ex/Em = 495/529 nm, and data were reported as the change in fluorescence after background subtraction.

### 4.6. Glutathione Fluorometric Assay

GSH, GSSG, and total glutathione were detected with the Biovision kit (K264-100) (Milpitas, CA, USA). A total of 2 × 10^5^ cells per well were seeded in a 6-well plate overnight and treated with YM155. Cells were collected with cell scraper and then prepared and measured following the manufacturer’s protocol. The GSH/GSSG ratio was normalized to the control group.

### 4.7. Cell Cycle Analysis

Cells were plated in 10 cm plates at 1 × 10^6^ cells per plate in 10 mL of culture medium at day 0. Fresh culture medium with YM155 or corresponding vehicle was added to each plate at day 1. Following treatment for 24 h, cells were harvested, fixed with cold 70% ethanol for at least 2 h at 4 °C, washed with PBS, and incubated in the dark with RNaseA (100 µg/mL) and propidium iodide (50 µg/mL) (Biolegend, San Diego, CA, USA) for 30 min at room temperature. Events were detected by flow cytometry (LSR II; BD Biosciences, East Rutherford, NJ, USA), with normalization and population selection. Data were analyzed using BD FACSDiva Software (BD Biosciences, Franklin Lakes, NJ, USA).

### 4.8. Apoptosis Analysis

Cell apoptosis was detected using the Alexa Fluor 647 Annexin V kit (Biolegend, San Diego, CA, USA), according to the manufacturer’s instructions.

### 4.9. Statistical Alanalysis

All experiments were repeated at least 3 times and data are presented as mean ± standard deviation (SD) unless otherwise indicated. All statistical analyses were performed in GraphPad Prism 8 (GraphPad Software Inc., San Diego, CA, USA). *p*-value less than 0.05 was considered statistically significant.

IC_50_ were calculated by AAT Bioquest, Inc., Sunnyvale, CA, USA. Quest Graph IC50 Calculator (v.1). Retrieved from https://www.aatbio.com/tools/ic50-calculator-v1 (accessed on 11 January 2021).

## 5. Conclusions

YM155 did not harm benign thyroid cells while inhibiting growth of all four ATC cell lines at 100 nM or lower, suggesting a high therapeutic window. YM155 induced DNA damage in ACT1 and THJ16T, and oxidative stress is at least partially responsible for increases in DNA damage. YM155-induced DNA damage in ACT1 and THJ16T led to cell cycle arrest and apoptosis. YM155 could be a candidate for ATC therapy, however, it remains unclear as to why some ATC cell lines respond better to YM155 than others. We are seeking to answer this question by identifying the molecular target of YM155 in ATC. Discovering specific targets of YM155 will advance an individualized approach to ATC treatment by identifying patients likely to respond to treatment.

## Data Availability

Not applicable.

## Supplementary Materials

The following are available online at https://www.mdpi.com/1422-0067/22/4/1961/s1.
