# Supplementary material for: Ym155 Induces Oxidative Stress-Mediated DNA Damage and Cell Cycle Arrest, and Causes Programmed Cell Death in Anaplastic Thyroid Cancer Cells
Source: Int J Mol Sci. 2021 Feb 16;22(4):1961. doi: 10.3390/ijms22041961 (PMC7920419; doi:10.3390/ijms22041961)
Supplement: Supplementary file 1 [file ijms-22-01961-s001.pdf]

International Journal of *Molecular Sciences*

Supplementary Tables

Table S1. RT-PCR primer sequences

	Gene	Forward (5'-3')	Reverse (5'-3')
**1**	18S rRNA	GTAACCCGTTGAACCCCATT	CCATCCAATCGGTAGTAGCG
**2**	TG	CTGGTGTGTCATGGACAGCGGAGAA	CCCGAGATTGTCTCACACAGGAT
**3**	TPO	ACAGCATCACTGGCATGAAC	GTGCACAAAGTCCCCATTCT
**4**	TSHR	CCCAGTACGCAGACTCTGAA	CCAGTGTTGAAAATGCCAAG
**5**	FOXE1	TGACCTGGGCTGGTTTTCC	TGCTGACGCACAGTTCAACTC
**6**	NKX2-1	CCATGAGGAACAGCGCCTC	CTCACGTCCCCCAGCGA
**7**	PAX8	CCTTTGTGAATGGCAGACCT	TAGGGAGGTTGAATGGTTGC
